# Enterocolic phlebitis: a rare cause of bowel ischemia and review of the literature

**DOI:** 10.1093/gastro/goad002

**Published:** 2023-02-01

**Authors:** Stefanie Bowee, Sophia B Matter, Heather Dawson, Roman A Inglin

**Affiliations:** Department of General Surgery, Spital Interlaken, Unterseen, Switzerland; Department of General Surgery, Spital Interlaken, Unterseen, Switzerland; Institute of Pathology, University of Bern, Bern, Switzerland; Department of General Surgery, Spital Interlaken, Unterseen, Switzerland

**Keywords:** phlebitis, acute abdomen, mesenteric vascular occlusion, mesenteric ischemia, colectomy, case report

## Abstract

Enterocolic phlebitis (EP) is a rare cause of bowel ischemia due to isolated venulitis of the bowel wall and mesentery without arterial involvement. EP is often misdiagnosed as inflammatory bowel disease, carcinoma, or diverticulitis due to non-specific symptoms as well as non-specific clinical and radiological findings. While unresponsive to pharmacotherapy, surgical resection of the affected bowel appears to be the only successful therapy with a very low recurrence rate. Etiology of EP remains unknown. We report a case of EP with rare presentation in the left hemicolon and unusual histological findings emphasizing the heterogeneity of this cause of enterocolic ischemia. The review and comparison of the three entities—EP, mesenteric inflammatory veno-occlusive disease (MIVOD), and idiopathic myointimal hyperplasia of mesenteric veins (IMHMV), all describing patterns of bowel ischemia due to isolated pathology of mesenteric veins—reveal that the current terminology is unclear. EP and MIVOD are very similar and may be considered the same disease. IMHMV, though, differs in localization, symptom duration, and histological findings but also shares features with EP and MIVOD. Further studies and harmonized terminology are inevitable for better understanding of the disease, prevention of unnecessary pharmacotherapy, and reduction in time to diagnosis.

## Introduction

Enterocolic phlebitis (EP) is a rare cause of bowel ischemia due to vasculitis of small veins of the bowel wall and mesentery in absence of arterial involvement or signs of systemic vasculitis. Any part of the bowel can be affected with predilection for the terminal ileum and the right hemicolon. Symptoms as well as radiologic and endoscopic appearances are often non-specific leading to clinical misdiagnosis (such as carcinoma, inflammatory bowel disease, or diverticulitis). Since EP shows no improvement with pharmacotherapy, surgical resection is the only successful therapy with a very low recurrence rate. To date, the etiology of EP remains unknown [1]. Other diseases of isolated phlebitis limited to the bowel have been described, namely mesenteric inflammatory veno-occlusive disease (MIVOD) and idiopathic myointimal hyperplasia of mesenteric veins (IMHMV). Since EP, MIVOD, and IMHMV show similar clinical and histological findings, they are sometimes used as synonyms but might be described as different subcategories of isolated enterocolic phlebitis.

A differential diagnosis of non-infectious vasculopathy of the gastrointestinal tract is systemic vasculitis (such as Behcet’s disease, Henoch–Schonlein purpura, or systemic lupus erythematosus), which may present first with gastrointestinal symptoms. Systemic vasculitis also rarely presents as single-organ vasculitis limited to the gastrointestinal tract. However, there are always both veins and arteries affected, and specific antibodies can usually be detected. Another disease spectrum that mainly affects the veins is veno-occlusive disease; there are mainly two eligible disease patterns: sinusoidal obstruction syndrome of the liver and pulmonary veno-occlusive disease.

We report the case of a rare presentation of EP in the left hemicolon associated with an unusual lack of necrosis and a predominant granulomatous component in the histological findings. We further performed a systematic review of the literature aiming at classifying the three subtypes EP, MIVOD, and IMHMV. The results of this publication shall contribute to a more standardized nomenclature within the realm of EP.

## Case presentation

In April 2020, an 81-year-old male was admitted to our hospital presenting with intermittent lower abdominal pain and associated recurrent fever for 11 days, as well as persistent anorexia and functional decline over the past month. Despite an empirical 5-day course of oral ciprofloxacin for presumed diverticulitis started earlier by the general practitioner, inflammation markers were permanently increased. No other gastrointestinal symptoms were reported. The past medical history of our patient was notable for coronary heart disease, chronic renal failure, and gout. He had been taking aspirin and pravastatin. Over-the-counter medications or herbal medicine were denied. An appendectomy had been performed in adolescence.

**Figure 1.**
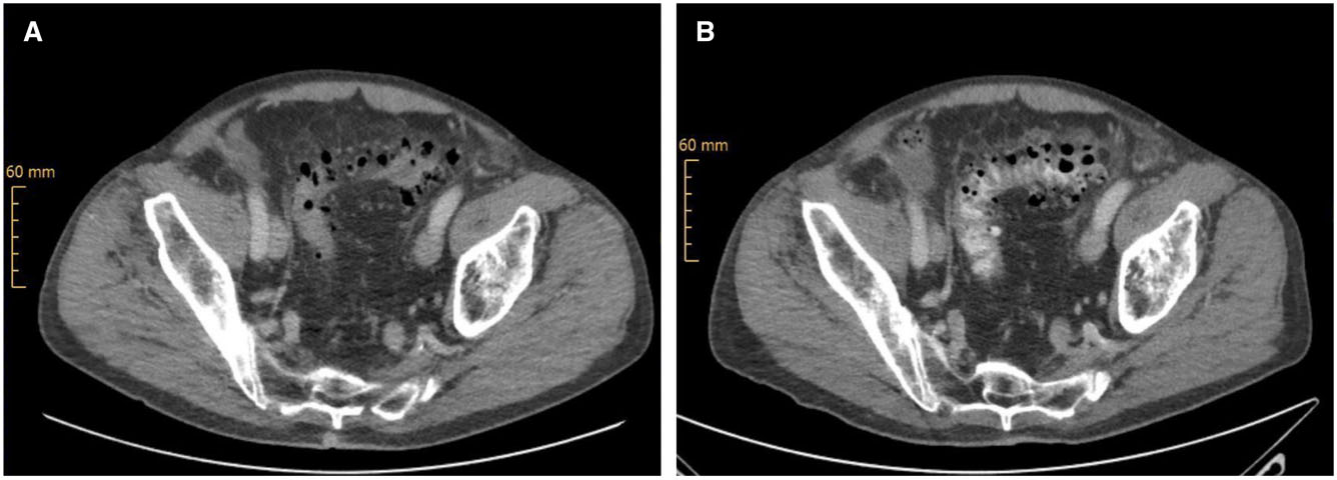
Computed tomography of the abdomen demonstrated wall thickening of the sigmoid and progressive pericolic fat stranding during hospitalization. (A) Imaging features at first presentation. (B) Imaging changes after 10 days of intravenous empiric antibiotic therapy.

Body temperature was 36.0°C, while a pulse rate of 80 beats per minute and blood pressure of 165/92 mmHg were measured. The abdominal examination revealed a peritonism of the lower left quadrant. Additionally, podagra on the right side was identified. The blood-examination results included a white blood cell (WBC) count of 11 × 10^9^/L (normal range 4.0–10.0 × 10^9^/L), a platelet count of 426 × 10^9^/L (150–400 × 10^9^/L), a C-reactive protein (CRP) level of 286 mg/L (<5 mg/L), and a serum creatinine level of 117 μmol/L (44–97 μmol/L). Other routine blood and urine tests were within the normal range. A computed tomography (CT) scan of the abdomen demonstrated marked colon diverticulosis, wall thickening of the sigmoid, and pericolic fat stranding (Figure 1). These findings were interpreted as acute sigmoid diverticulitis.

Escalation of the empiric antibiotic therapy to piperacillin-tazobactam led to a reduction in the abdominal discomfort. However, the WBC and CRP levels remained almost unchanged at high levels, even after changeover of the antibiotic therapy to meropenem. Consequently, another CT scan, done on Day 10 of hospitalization, showed progressive pericolic fat stranding surrounding the transverse and sigmoid colon (Figure 1).

**Figure 2.**
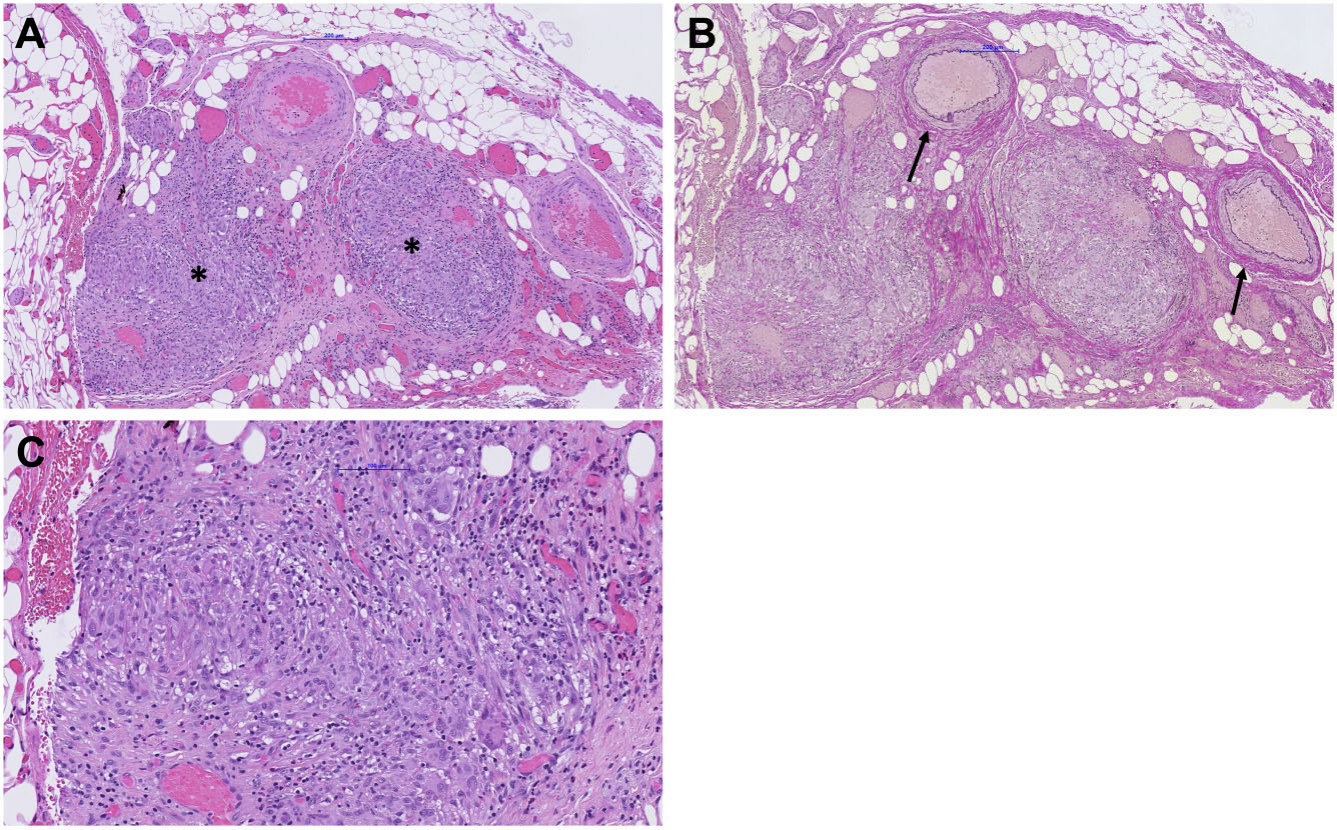
Histopathologic findings. (A) Hematoxylin and eosin stain reveals enterocolic phlebitis with significant occlusion of the lumen of medium-sized venous vessels (asterisks). (B) Elastica van Giesson stain reveals destruction of the elastic lamina and intact accompanying arterioles (arrows). (C) Closer inspection of the inflammatory infiltrate shows a predominant non-necrotizing granulomatous component accompanied by lymphocytes, eosinophils, and plasma cells.

Given the lack of an adequate clinical and biological response to antibiotic treatment, diagnostic laparoscopy with possible extension of the surgery depending on intraoperative findings was indicated. Patient consent was obtained accordingly prior to surgery. A firm sigmoid colon mass adherent to the anterolateral abdominal wall was found intraoperatively. A laparoscopic left-sided hemicolectomy with complete mesocolic excision and primary colorectal anastomosis was performed and the specimen submitted for pathological examination. Grossly, an induration of the mesocolic fat and diverticulosis were described. Histologic findings revealed a pronounced granulomatous, non-necrotizing obliterative phlebitis affecting small- to medium-sized venous vessels with admixed eosinophils, plasma cells, and lymphocytes (Figure 2). Remarkably, the overlying mucosa was intact without signs of ischemia. There was no evidence of venous thrombi. Furthermore, no storiform fibrosis was found and immunostains for IgG and IgG4 did not reveal an increase in IgG4-positive plasma cells. The histological pattern of obliterative phlebitis without affection of the arteries was suggestive of enterocolic phlebitis entailing the diagnosis of EP.

The perioperative antibiotic therapy with meropenem was continued for 14 days and, with respect to the histologic findings of a non-necrotizing vasculitis, steroids with a tapering regimen were initiated.

The post-operative course was uneventful and the patient left the hospital 19 days after admission and 9 days after surgery. Rheumatologic tests did not show any signs of systemic vasculitis while antinuclear antibody and antineutrophil cytoplasmic antibody screenings were negative. A follow-up colonoscopy after 6 weeks did not identify any pathology. The patient remained asymptomatic at 6 weeks as well as at the 7- and 17-month follow-ups.

## Literature review

While screening the literature for data about EP, it was noticed that the most commonly used terms EP, MIVOD, and IMHMV are inconsistently used—in some cases as different entities, in others as synonyms. In order to get a better overview of the published data and to classify these three terms more accurately, we performed a systematic review of the pertinent literature dealing with the classification of EP, MIVOD, and IMHMV with respect to patient characteristics, clinical presentation, symptom duration, predominantly affected bowel segments, and histological findings, and thereby focused on case reports and case series. The primary goals of this literature review were an accurate classification of our case into one of the three existing disease patterns as well as a critical analysis and discussion of the available data addressing the main points of controversy of EP. Pooling of original raw data and separate analysis were not done.

## Methods

A literature search in English- and German-language publications (abstract always in English) was performed using the National Library of Medicine (PubMed) database. The free-text queries included the keywords “mesentery veno-occlusive disease,” “myointimal hyperplasia mesenteric veins,” and “enterocolic phlebitis.” Links to related articles and cross-reading of citations in related articles were surveyed. Keywords were combined to narrow or widen the search. Based on the title and abstract of the publication, full articles were either downloaded or requested through this institution’s library. The publications were assessed as eligible when they included a case report and the described cases were clearly assigned to either EP, MIVOD, or IMHMV, and when they met the following quality criteria: they should include patient characteristics (gender and age), symptoms, symptom duration, localization of the venous changes and details of the histological findings such as venous or arterial changes, involved inflammatory cells, exact localization of the phlebitis, and remarks on ischemia and necrosis of the bowel. Each of the quality criteria was, if present, rated with a star, with a maximum of 5 stars possible per valued publication. Publications using different terms than EP, MIVOD, or IMHMV; those whose full text version could not be retrieved; and those with a quality rating of <3 stars were excluded from further analysis.

## Results

**Figure 3.**
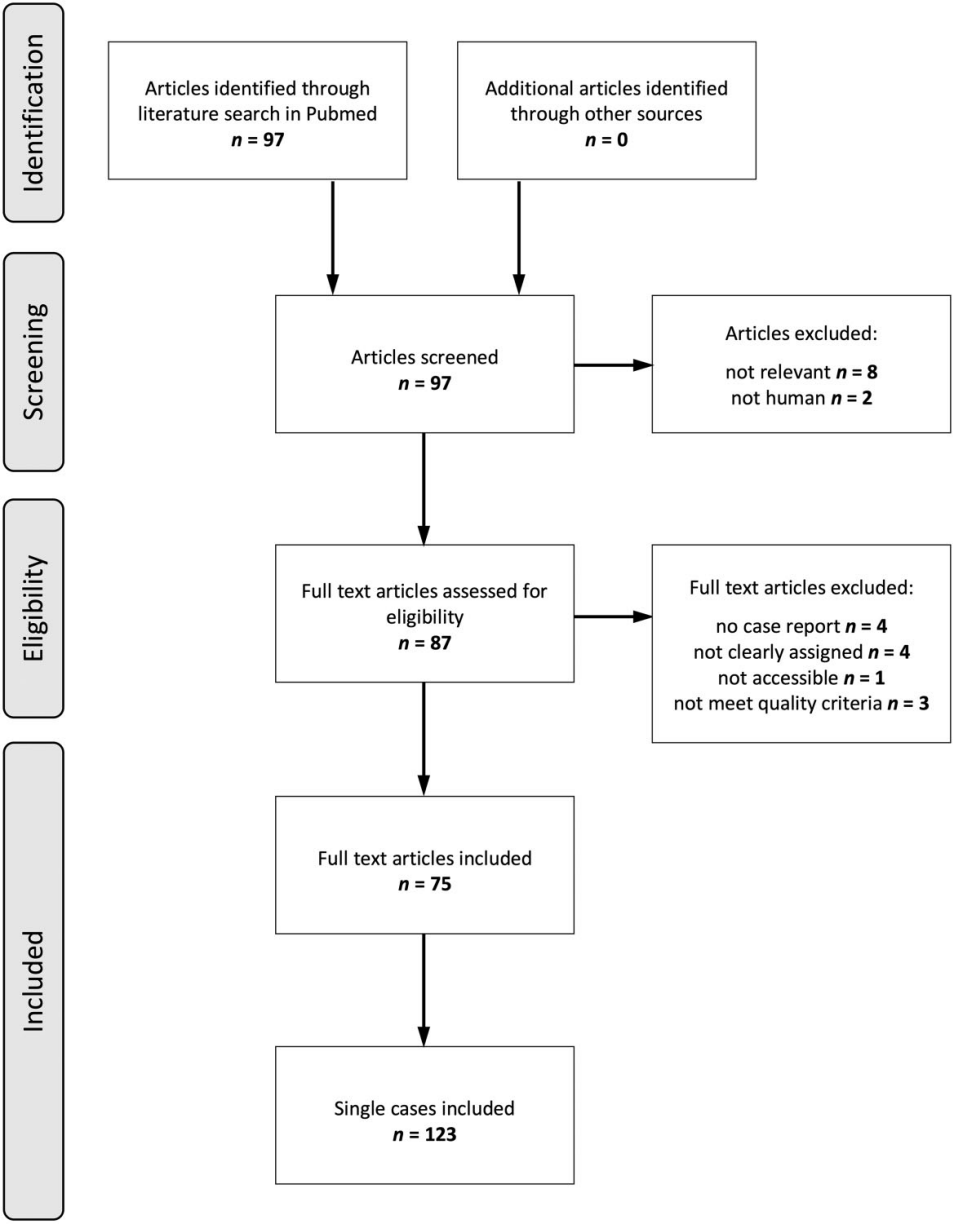
PRISMA flow diagram of the article-selection process

The literature search yielded a total of 97 publications published from 1976 up to May 2021. Upon applying inclusion and exclusion criteria, a total of 22 publications were excluded. The detailed review process is shown in Figure 3. We finally extracted and analysed 33 case reports of EP, 41 of MIVOD, and 49 of IMHMV. The extracted data are shown in Supplementary Table 1.

### Clinical features

**Figure 4.**
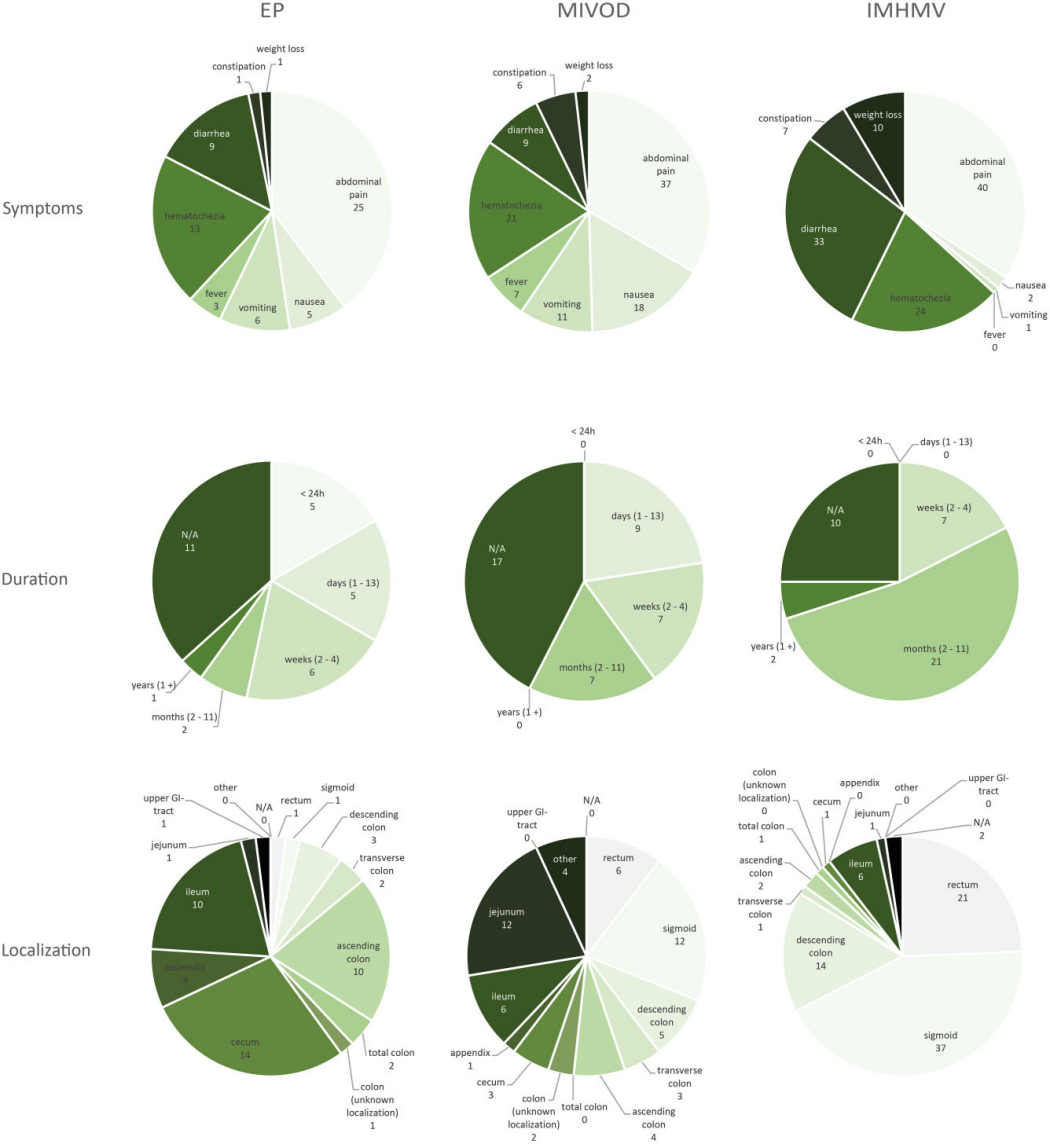
Clinical features of the reviewed cases of EP, MIVOD, and IMHMV. EP, enterocolic phlebitis; MIVOD, mesenteric inflammatory veno-occlusive disease; IMHMV, idiopathic myointimal hyperplasia of mesenteric veins; N/A, not available.

**Figure 5.**
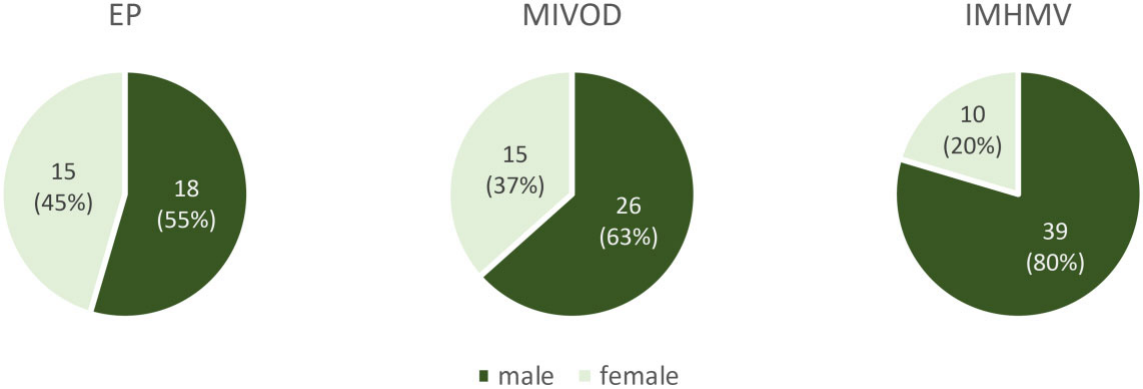
Gender distribution of the reviewed cases of EP, MIVOD, and IMHMV. EP, enterocolic phlebitis; MIVOD, mesenteric inflammatory veno-occlusive disease; IMHMV, idiopathic myointimal hyperplasia of mesenteric veins.

EP was first described as “enterocolic lymphocytic phlebitis” in 1989 by Saraga *et al*. [2]. The authors stated that this form of isolated EP could represent a spectrum of histological features, including a granulomatous component. Consequently, they later proposed to use the more inclusive term “enterocolic phlebitis” [3]. The clinical presentation of EP is unspecific. Patients normally present with moderate to severe abdominal pain, diarrhea, and hematochezia, often referred to medical services for suspicion of an acute abdomen. However, there were also asymptomatic cases of EP examined for anemia [4, 5] or treated for a colon carcinoma [6]. Symptom duration ranges from a few hours to several months, but frequently patients present within a few days of onset. The phlebitis can occur in any part of the bowel, but mostly involves the right hemicolon, the cecum, and the ileum. Both sexes are equally affected and, even though EP appears in all ages, the disease seems to predominantly occur in patients >50 years of age (median age 60 years). MIVOD, first described by Flaherty *et al.* in 1994 [7], has a very similar presentation to EP regarding the characteristics of patients (median age 52.5 years) and duration of symptoms. Yet, MIVOD predominantly occurs in the descending colon and the sigmoid, and affected patients more commonly suffer from nausea and vomiting. In comparison, patients presenting with IMHMV usually complain of a longer period of symptoms of up to several months. Further, IMHMV shows a clear predominance in male patients and most often affects the left colon favoring the sigmoid. This obviously distinguishes IMHMV from EP and MIVOD. IMHMV, first described 1991 by Genta *et al*. [8], was thought to occur mainly in young patients, although review of the literature shows a median age of 62 years (mean age 59 years) at diagnosis, which is similar to EP and even higher than the median age of MIVOD patients. Figures 4 and 5 summarize the patient characteristics and clinical pictures of the analysed cases. No data are available on the mortality of EP, MIVOD, and IMHMV. In one case of MIVOD [9] and three cases of IMHMV [10–12], bowel perforation was described—a complication surely leading to a higher morbidity and an increased risk of mortality. In our review, there was no reported case of death associated with the disease itself.

### Histological characteristics

The characteristic histological pattern of EP is a lymphocytic perivenular and venular wall infiltration affecting veins of the bowel wall and mesentery, particularly the submucosal veins of small size (<2 mm). Sometimes, granulomatous and necrotizing phlebitis is found [3]. Additionally venous thrombi of different ages are usually present resulting in venous congestion with edema and tissue compression, probably being the reason why EP is frequently associated with intestinal ischemia [1]. Interestingly, arteries are always unaffected.

The histological features of MIVOD with predominantly lymphocytic phlebitis of the bowel wall and mesentery accompanied by venous thrombi of different ages and necrotic mucosal lesions [7] are barely distinguishable from those of EP.

While sharing the histological basics of intestinal ischemia due to venous occlusion without affection of arteries, IMHMV clearly differs from EP and MIVOD, since the former does not show evidence of inflammatory cells. Instead there is typically a myointimal hyperplasia present due to proliferation of smooth muscle cells resulting in non-thrombotic occlusion or stenosis of mesenteric veins [8]. Due to the heterogeneity of information about histologic findings in the reviewed publications, further differentiation was not reasonable.

### Diagnostic findings

To date, no characteristic preoperative diagnostic features are known. Patients with EP, MIVOD, or IMHMV often present with leukocytosis and elevated CRP levels, but there are no specific laboratory markers. Vasculitis-associated antibodies are not detectable. The radiologic pattern is unspecific with either wall thickening of the affected bowel part and stranding of the adjacent fatty tissue [9, 13, 14] or evidence of a mass [15]. Colonoscopy findings range from mucosal edema to marked inflammatory changes with mucosal ulceration and luminal stenosis. Mucosal biopsy results usually reveal non-specific mucosal inflammation or ischemic changes leading to misinterpretation as chronic inflammatory bowel disease or ischemic colitis [16–18].

## Discussion

This case report presents a rare and poorly characterized disease of localized phlebitis of the colon. The unspecific clinical presentation as well as unspecific radiologic and endoscopic findings often lead to misdiagnosis of chronic inflammatory bowel disease, ischemic colitis, or tumor. In the literature, only few data about isolated phlebitis of the bowel are available and nearly all publications are case reports except from a few non-systematic reviews. There is no uniform nomenclature. Therefore, we tried to methodically classify EP, MIVOD, and IMHMV. Here it has become evident, from a clinical and histological point of view, that EP and MIVOD may be considered the same disease and we therefore suggest summarizing them under the short and descriptive term “enterocolic phlebitis.” IMHMV shows, however, distinct differences, enabling a differentiation from EP. There are for instance no histological signs of acute phlebitis. The lack of an inflammatory infiltration might also be interpreted as a chronic change, leaving it up for debate whether IMHMV might be a form of chronic EP [13], where histological findings in some cases also include myointimal hyperplasia [19, 20]. However, considering the clearly different histological pattern along with the differences in clinical features such as longer symptom duration, clear predominance in male patients, and mainly affecting the sigmoid colon, IMHMV seems to be distinguishable from EP.

In our case, patient characteristics as well as the aspect and duration of symptoms are consistent with the disease pattern of EP. Nevertheless, the histology revealed rather unusual features. It demonstrated pronounced occlusive non-necrotic phlebitis. Remarkably, there was no evidence of mucosal ischemia, although most cases of EP present with intestinal infarction [1, 21]. The absence of venous thrombi might explain the lack of ischemic changes to the mucosa in our patient. Furthermore, the mainly granulomatous infiltration of venous walls and the perivenous tissue is only described in a minority of EP cases. A review on EP and its clinicopathologic features revealed that only 6 out of 24 patients had predominantly granulomatous phlebitis [1]. These findings emphasize the heterogeneity of this disease. The spectrum of histological findings makes accurate categorization challenging and more detailed histological data are necessary to find out whether EP and IMHMV are subcategories of the same disease or different entities.

The etiology of EP and IMHMV is still unclear. Signs of systemic vasculitis are absent, in contrast to diseases featuring systemic vasculitis with gastrointestinal tract involvement. Further, IMHMV with non-thrombotic venous occlusion in absence of acute inflammatory changes is reminiscent of the veno-occlusive diseases sinusoidal obstruction syndrome and pulmonary veno-occlusive disease. Sinusoidal obstruction syndrome occurs mostly as a complication after hematopoietic stem-cell transplantation or high-dose chemotherapy and is thought to be a toxic injury to endothelial cells. Pulmonary veno-occlusive disease, with vascular lesions of veins, capillaries, and arteries, is also often related to chemotherapeutic agents but here a genetic mutation plays a central role in the pathogenesis [22]. Our literature review reveals only one patient with previous stem-cell transplantation [23]. Sporadic cases of sinusoidal obstruction syndrome after consumption of herbal remedies with toxic alkaloids have been reported. But even here we only found one patient with IMHMV taking Chinese herbals [24]. Whether IMHMV is related with veno-occlusive diseases remains unclear. Overall, the review did not show any obvious medications or previous health conditions facilitating EP or IMHMV. There are even several previously healthy patients affected without previous medication. Moreover, some case reports have linked EP to IgG4-related disease (IgG4-RD) [5, 25]. However, diagnostic criteria for IgG4-RD have been revised as its characterization has improved over the years [26] and currently include a combination of clinical, radiological, serological, and histopathological parameters to distinguish between definite, probable, and possible IgG4-RD. In our case, diagnostic criteria of IgG4-RD were not present. However, this does not preclude that a subset of EP cases may represent a manifestation of IgG4-RD when diagnostic criteria are fulfilled. In summary, although there are similarities to other diseases where a possible relationship is not excluded, solid evidence regarding the cause of this single-organ venulitis or venulopathy is still lacking.

Due to the clinical resemblance to chronic inflammatory bowel disease or diverticulitis, patients with EP are often inaccurately treated with steroids or antibiotics. In our case, the primary diagnosis of presumed diverticulitis entailed unsuccessful treatment with broad-spectrum antibiotics. Absence of clinical and biological improvement prompted the indication for surgery that led to the correct diagnosis. This is likely to be the usual clinical course of most EP and IMHMV cases. Due to the poor response of EP and IMHMV to pharmacotherapy, the only current effective therapy is resection of the involved bowel segment. There are only very few reported cases of disease recurrence after surgical treatment, even though in several cases phlebitis was present at the resection margins [3]. We only found one case of EP [2] and one of MIVOD [27] with histologically proven recurrence. Another case of clinical recurrence of MIVOD without histological work-up was reported [28]. The fact that most of the patients remain disease-free after surgery corresponds to our patient who is in good health and free of recurrence ≥17 months after hemicolectomy. The steroid treatment we initiated once the histological findings were available might not have been necessary considering that several case reports describe no clinical effect of corticosteroids [18, 29, 30] and in most cases patients remained recurrence-free without pharmacotherapy after surgical treatment. There are no data on whether anticoagulation influences EP since a thrombotic cause is usually not suspected based on the clinical presentation.

## Conclusions

EP, MIVOD, and IMHMV are currently poorly understood diseases that remain a diagnostic challenge. Patient history, symptoms, as well as clinical and radiological findings are non-specific, leading to clinical misdiagnosis and unnecessary medical treatment. There may be many unreported cases with oligosymptomatic or subclinical course because there is currently no possibility of preoperative diagnosis. EP, MIVOD, and IMHMV are by nature histological diagnoses characterized by a spectrum of alterations in veins and venules but not in arteries, often leading to localized bowel ischemia. Our case emphasizes the heterogeneity in histological findings of EP with a rare presentation of granulomatous infiltration and an unusual absence of mucosal ischemia. Unsuccessful clinical and/or biological improvement under presumably adequate pharmacotherapy within due time should prompt the indication for surgical resection of the involved bowel segment. Surgery represents the therapy of choice of EP, MIVOD, and IMHMV, providing excellent long-term results.

Given the rarity of the disease and its heterogeneity of clinical and histomorphological presentations, it seems difficult to gather more valid than retrospective data. However, standardization of the nomenclature might help to more accurately classify the published cases. We suggest summarizing EP and MIVOD, which seem to be the same disease, under the term EP. Publication of cases and review of the current literature might further help to eventually better understand the disease and its underlying causes, which in return may give rise to new diagnostic tools facilitating accurate and prompt diagnosis as well as indication for surgery. This would avoid prolonged symptom duration and possibly prevent inadequate pharmacotherapy.

## Supplementary Material

goad002_Supplementary_Data
