# Supplementary material for: Enterocolic phlebitis: a rare cause of bowel ischemia and review of the literature
Source: Gastroenterol Rep (Oxf). 2023 Feb 1;11:goad002. doi: 10.1093/gastro/goad002 (PMC9891246; doi:10.1093/gastro/goad002)
Supplement: goad002_Supplementary_Data [file goad002_supplementary_data.docx]

**Table 1.** Summary Table of included Studies

| Author | Year | Patient characteristics | |  | Symptoms | | | | Localization | Histology | | | | | | | | | | | | | Quality rating |
| --- | --- | --- | --- | --- | --- | --- | --- | --- | --- | --- | --- | --- | --- | --- | --- | --- | --- | --- | --- | --- | --- | --- | --- |
|  |  |  |  |  |  |  |  |  |  | Veins | | | | | | Arteries | Thrombosis | Bowel ischemia | | Bowel necrosis | Cell differentiation of inflammatory infiltrate | Granulomatous inflammation |  |
|  |  | Gender | Age, year |  | Quality | Special features of clinical presentation | Duration at first medical presentation | Time to surgery |  | Size | Location of venous lesions | Myointimal hyperplasia | Focal necrosis | Infiltration of the wall | Luminal stenosis |  |  |  |  |  |  |  |  |
| **EP** | | | | | | | | | | | | | | | | | | |  | | | | |
| Saraga | 1989 | M | 58 |  | Vomiting, fever, abdominal pain, hematochezia | Acute abdomen | 2 weeks | N/A | Ascending and transverse colon | all sizes | Mostly submucosal | N/A | Yes | Yes | N/A | Unaffected | Thrombi of different age | N/A | | Mostly mucosa, foci of full thickness necrosis | Lymphocytes, plasma cell | N/A | ***** |
| Saraga | 1989 | F | 77 |  | Abdominal pain | Acute abdomen | 10 days | 3 weeks | Ileum | all sizes | Mostly submucosal | N/A | Yes | Yes | N/A | Unaffected | Thrombi of different age | N/A | | Mostly mucosa, foci of full thickness necrosis | Lymphocytes, plasma cell | N/A |  |
| Saraga | 1989 | M | 66 |  | Abdominal pain, nausea, hematochezia | Acute abdomen | 2 days | N/A | Ileum | all sizes | Mostly submucosal | N/A | Yes | Yes | N/A | Unaffected | Thrombi of different age | N/A | | Mostly mucosa, foci of full thickness necrosis | Lymphocytes, plasma cell | N/A |  |
| Haber | 1993 | F | 78 |  | Abdominal pain, hematochezia, diarrhea | N/A | 3 weeks | N/A | Cecum | Veins, venules | Submucosa, subserosa, extramural | N/A | Yes | Yes | Yes | Unaffected | Thrombi of different age | Yes | | Ischemic necrosis of mucosa | Lymphocytes, neutrophils, eosinophils, single giant cell | N/A | ***** |
| Chergui | 1997 | M | 65 |  | Abdominal pain, fever, diarrhea | N/A | < 24 h | N/A | Cecum | Small, medium, large | Submucosa | No | No | Yes | No | Unaffected | Subacute thrombi | N/A | | Submucosal necrosis | Lymphocytes, giant cell | N/A | ***** |
| Saraga | 2000 | M | 67 |  | Abdominal pain, weight loss | Acute abdomen | 4 weeks | N/A | Ileum | All sizes | Mostly mucosal and submucosal | N/A | Yes | Yes | N/A | Unaffected | Acute thrombi | Yes | | Acute ischemic necrosis | Mainly neutrophiles, lymphocytes | N/A | ***** |
| Saraga | 2000 | F | 70 |  | Abdominal pain, fever, hematochezia | N/A | Several years | N/A | Transverse colon | All sizes | Mostly mucosal and submucosal, intramuscular | Yes | No | Yes | N/A | Unaffected | Thrombi of different age | Yes | | Chronic ischemic necrosis | Lymphocytes | Yes |  |
| Saraga | 2000 | F | 72 |  | Other (acute abdomen) | Acute abdomen | < 24 h | N/A | Ileum | All sizes | Mostly mucosal and submucosal | Yes | Yes | Yes | N/A | Unaffected | Thrombi of different age | Yes | | Acute and chronic ischemic necrosis | Lymphocytes, neutrophiles | Yes |  |
| Tuppy | 2000 | F | 74 |  | Abdominal pain | N/A | N/A | N/A | Ascending colon, cecum | N/A | Submucosa, subserosa | N/A | Yes | Yes | Yes | Unaffected | Yes | Ischemic colitis | | Transmural coagulation necrosis | Lymphocytes | N/A | **** |
| Arain | 2002 | M | 65 |  | Abdominal pain, nausea, vomiting, hematochezia, diarrhea | Abdominal mass | 2 weeks | N/A | Total colon | N/A | Colon wall and mesentery | N/A | N/A | Yes | N/A | Unaffected | N/A | Ischemic-type mucosa ulceration | | Fat necrosis of mesentery | Lymphocytes, eosinophiles | N/A | ***** |
| Pares | 2003 | F | 50 |  | Hematochezia | Haemodynamic instability | < 24 h | < 24 h | Ascending colon, cecum | Venules | Submucosa | Yes | N/A | Yes | Yes | Unaffected | N/A | N/A | | N/A | Lymphocytes | N/A | ***** |
| Abraham | 2004 | M | 68 |  | Abdominal pain, other (melena, hematemesis) | N/A | N/A | 9 months | Upper GI-tract, duodenum | Small, medium | Submucosa, subserosa, muscularis propria | N/A | No | Yes | Yes | Unaffected | Rare | N/A | | N/A | Lymphocytes, giant cells, histiocytes, plasma cells, eosinophils | Yes | ***** |
| Shiraki | 2004 | M | 73 |  | Abdominal pain, diarrhea, other (fatigue) | Abdominal mass | 3 weeks | 3 months | Descending colon | N/A | Submucosa, muscularis propria, fat tissue | N/A | Yes | Yes | Yes | Unaffected | N/A | N/A | | No | Lymphocytes | N/A | ***** |
| Wright | 2004 | M | 53 |  | Abdominal pain, vomiting, hematochezia, diarrhea | Abdominal mass | < 24 h | N/A | Ascending colon, cecum, appendix, ileum | N/A | Submucosa, muscularis propria, subserosa, mesentery | No | Yes | Yes | Yes | Unaffected | Thrombi of different age | Yes | | Hemorrhagic transmural necrosis at the cecum | Lymphocytes, plasma cells, eosinophils, histiocytes | N/A | ***** |
| Medlicott | 2006 | M | 37 |  | Abdominal pain, nausea, vomiting, hematochezia, diarrhea | N/A | < 24 h | N/A | Ascending colon, cecum, appendix | N/A | Submucosa, subserosa | N/A | Yes | N/A | Yes | Unaffected | Yes | Segmental ischemic colitis of cecum | | N/A | Lymphocytes, histiocytes, neutrophiles | N/A | ***** |
| Massasso | 2007 | F | 54 |  | Abdominal pain | N/A | 2 days | N/A | Ileum, cecum, ascending colon | Veins, venules | Submucosa, bowel wall, mesentery fat | N/A | N/A | N/A | N/A | Unaffected | Yes | N/A | | Hemorrhagic mucosal necrosis | Mixed infiltrate, lymphocytes | N/A | ***** |
| Massasso | 2007 | M | 50 |  | Abdominal pain | N/A | < 24 h | N/A | Cecum | Venules | Submucosa, muscularis mucosae | N/A | Yes | Yes | N/A | N/A | Yes | N/A | | Focal mucosal | Lymphocytes, neutrophiles | N/A |  |
| Jain | 2008 | M | 73 |  | Asymptomatic (iron deficiency anemia) | N/A | N/A | N/A | Ascending colon | N/A | Submucosa | No | No | Yes | N/A | Unaffected | No | No | | No | Lymphocytes, eosinophiles | No | **** |
| Cruz | 2009 | F | 29 |  | Abdominal pain | N/A | Long standing | N/A | Ascending colon, cecum, ileum | Small, medium | Submucosa | Yes | N/A | Yes | Yes | Unaffected | Thrombi of different age | Submucosal ischemia | | N/A | Lymphocytes, neutrophils, eosinophils, giant cells, histiocytes | No | **** |
| Nallamothu | 2011 | M | 29 |  | Abdominal pain, hematochezia | N/A | 3 days | N/A | Ascending colon, cecum, appendix, ileum | N/A | Submucosa | N/A | N/A | Yes | N/A | N/A | Yes | N/A | | Mucosal necrosis of ileocecal valve | Lymphocytes | N/A | **** |
| Galazka | 2012 | M | 35 |  | Abdominal pain, vomiting, diarrhea | N/A | 2 days | N/A | Ascending colon, cecum | small | Submucosa, muscularis propria, subserosa | Yes | Yes | Yes | Yes | Unaffected | Yes | Yes | | Transmural, ischemic and hemorrhagic necrosis | Lymphocytes, histiocytes, giant cells | N/A | ***** |
| Ghersin | 2013 | M | 37 |  | Abdominal pain | N/A | < 24 h | N/A | Cecum | N/A | Submucosa, subserosa | N/A | Yes | Yes | N/A | N/A | N/A | Colonic mucosa with ischemic-like changes | | N/A | Lymphocytes | N/A | ***** |
| Gomez | 2013 | F | 60 |  | Hematochezia | N/A | N/A | N/A | Colon (unknown localization) | N/A | N/A | Yes | Yes | Yes | Yes | Unaffected | N/A | N/A | | N/A | Lymphocytes | N/A | *** |
| Comtesse | 2014 | F | 20 |  | Other (acute abdomen) | Acute abdomen | N/A | N/A | Cecum, appendix | N/A | Submucosa, adventitia | N/A | N/A | Yes | Yes | Unaffected | N/A | N/A | | N/A | Lymphocytes, plasma cells (IgG4-positive) | Yes | ***** |
| Huiberts | 2014 | F | 42 |  | Abdominal pain, nausea, other (night sweats, hot flashes) | Abdominal mass | Several weeks | N/A | Jejunum | N/A | N/A | N/A | N/A | N/A | Yes | N/A | N/A | N/A | | N/A | Lymphocytes | N/A | *** |
| Laco | 2015 | F | 65 |  | Asymptomatic (anemia) | N/A | N/A | N/A | ascending colon | Small veins and venules | Submucosa, adventitia | N/A | No | Yes | Yes | Unaffected | Fresh thrombi | N/A | | Partial mucosal necrosis | Lymphocytes, plasma cells | No | **** |
| Louie | 2018 | F | 55 |  | Abdominal pain, constipation | N/A | N/A | N/A | Descending colon | N/A | N/A | N/A | N/A | N/A | N/A | N/A | N/A | N/A | | N/A | N/A | N/A | *** |
| Louie | 2018 | F | 56 |  | Abdominal pain | N/A | N/A | N/A | Descending, transverse and ascending colon | N/A | N/A | N/A | N/A | N/A | N/A | N/A | N/A | N/A | | N/A | N/A | N/A |  |
| Louie | 2018 | M | 81 |  | Asymptomatic (carcinoma) | N/A | N/A | N/A | Descending colon | N/A | N/A | N/A | N/A | N/A | Yes | N/A | N/A | N/A | | N/A | Inflammatory and spindle cells | N/A |  |
| Louie | 2018 | M | 17 |  | Abdominal pain, hematochezia | Acute abdomen | N/A | N/A | Cecum, ileum | N/A | N/A | N/A | N/A | N/A | N/A | N/A | N/A | N/A | | N/A | N/A | N/A |  |
| Mora-Guzman | 2018 | F | 60 |  | Abdominal pain, hematochezia, diarrhea | N/A | N/A | N/A | Total colon | N/A | Mucosa, submucosa | N/A | Yes | Yes | N/A | N/A | N/A | Transmural ischemic lesions | | Necrotic areas in the mucosa | Lymphocytes, neutrophils | N/A | *** |
| Nakaya | 2019 | M | 62 |  | Abdominal pain | N/A | 3 months | 4 months | Ileum | 200–1000 µm | Subserosa | Yes | No | Yes | Yes | Unaffected | No | N/A | | N/A | Lymphocytes, neutrophils | N/A | ***** |
| Farber | 2021 | M | 65 |  | Abdominal pain, nausea, vomiting, hematochezia, diarrhea | N/A | Several months | N/A | Rectum, sigmoid colon | N/A | Submucosa | Yes | Yes | Yes | N/A | Unaffected | N/A | N/A | | N/A | Lymphocytes | N/A | ***** |
| **MIVOD** | | | | | | | | | | | | | | | | | | |  | | | | |
| Flaherty | 1994 | F | 78 |  | Abdominal pain, nausea | N/A | 4 weeks | N/A | Sigmoid colon | N/A | N/A | Yes | No | Yes | N/A | Unaffected | Thrombi of different age | Yes | | Mostly mucosa, parts with transmural necrosis | Lymphocytes, histiocytes, giant cells | Yes | ***** |
| Flaherty | 1994 | M | 34 |  | Abdominal pain, nausea, vomiting | N/A | 3 weeks | N/A | Jejunum | N/A | N/A | No | Yes | Yes | N/A | Unaffected | Thrombi of different age | Yes | | Mostly mucosa, parts with transmural necrosis | Neutrophils | No |  |
| Flaherty | 1994 | F | 27 |  | Abdominal pain, nausea, vomiting, hematochezia | N/A | 7 days | N/A | Jejunum | N/A | N/A | No | Yes | Yes | N/A | Unaffected | Thrombi of different age | Yes | | Mostly mucosa, parts with transmural necrosis | Neutrophils | No | ***** |
| Flaherty | 1994 | M | 46 |  | Other (colitis of unknown cause) | N/A | Other (several months) | N/A | Sigmoid colon | N/A | N/A | Yes | No | Yes | N/A | Unaffected | Thrombi of different age | Yes | | Mostly mucosa, parts with transmural necrosis | Lymphocytes | No |  |
| Flaherty | 1994 | F | 36 |  | Abdominal pain | N/A | 2 weeks | N/A | Ascending colon | N/A | N/A | No | No | Yes | N/A | Unaffected | Thrombi of different age | Yes | | Mostly mucosa, parts with transmural necrosis | Lymphocytes | No |  |
| Flaherty | 1994 | F | 60 |  | Abdominal pain | N/A | 3 weeks | N/A | Ascending colon | N/A | N/A | Yes | No | Yes | N/A | Unaffected | Thrombi of different age | Yes | | Mostly Mucosa, parts with transmural necrosis | Lymphocytes | No |  |
| Flaherty | 1994 | M | 35 |  | Abdominal pain, hematochezia | N/A | 4 weeks | N/A | Sigmoid colon | N/A | N/A | No | No | Yes | N/A | Unaffected | Thrombi of different age | Yes | | Mostly mucosa, parts with transmural necrosis | Lymphocytes | No |  |
| Gül | 1996 | M | 24 |  | Abdominal pain, hematochezia, diarrhea, other (purpuric skin lesions) | N/A | 2 months | N/A | Jejunum | All sizes | N/A | N/A | yes | Yes | N/A | Unaffected | Thrombi of different age | N/A | | Hemorrhagic necrosis of bowel wall | Lymphocytes | N/A | ***** |
| Ailani | 1997 | M | 29 |  | Abdominal pain, vomiting, fever, hematochezia, diarrhea | N/A | 5 days | N/A | Ileum, jejunum | Venules | Submucosal, mesenteric | N/A | N/A | N/A | N/A | Unaffected | Thrombo-phlebitis, mesenteric vein thrombosis | Ischemic enteritis | | N/A | N/A | N/A | **** |
| Lie | 1997 | F | 65 |  | Abdominal pain, nausea, vomiting, hematochezia | Acute abdomen | N/A | N/A | Jejunum | N/A | N/A | N/A | N/A | Yes | N/A | Unaffected | N/A | Yes | | N/A | N/A | Yes | *** |
| Lie | 1997 | F | 76 |  | Abdominal pain, nausea, vomiting, hematochezia | Acute abdomen | N/A | N/A | Jejunum | N/A | N/A | N/A | N/A | Yes | N/A | Unaffected | N/A | Yes | | N/A | N/A | Yes |  |
| Lie | 1997 | M | 46 |  | Abdominal pain, nausea, vomiting, hematochezia | Acute abdomen | N/A | N/A | Colon (unknown localization) | N/A | N/A | N/A | N/A | Yes | N/A | Unaffected | N/A | Yes | | N/A | Lymphocytes | No |  |
| Lie | 1997 | M | 49 |  | Abdominal pain, nausea, vomiting, hematochezia | Acute abdomen | N/A | N/A | Jejunum | N/A | N/A | N/A | N/A | Yes | N/A | Unaffected | N/A | Yes | | N/A | Lymphocytes | No |  |
| Lie | 1997 | M | 59 |  | Abdominal pain, nausea, vomiting, hematochezia | Acute abdomen | N/A | N/A | Jejunum | N/A | N/A | N/A | N/A | Yes | N/A | Unaffected | N/A | Yes | | N/A | Lymphocytes | No |  |
| Lie | 1997 | M | 39 |  | Abdominal pain, nausea, vomiting, hematochezia | Acute abdomen | N/A | N/A | Colon | N/A | N/A | N/A | N/A | Yes | N/A | Unaffected | N/A | Yes | | N/A | N/A | No |  |
| Lie | 1997 | M | 68 |  | Other (recurrent acalculous cholecystitis) | N/A | N/A | N/A | Other (gallbladder) | N/A | N/A | N/A | N/A | Yes | N/A | Unaffected | N/A | N/A | | N/A | Lymphocytes | No |  |
| Lie | 1997 | M | 61 |  | Abdominal pain, nausea, vomiting, hematochezia | Acute abdomen | N/A | N/A | Other (omentum) | N/A | N/A | N/A | N/A | Yes | N/A | Unaffected | N/A | Yes | | N/A | Lymphocytes | No |  |
| Lie | 1997 | F | 42 |  | Abdominal pain, nausea, vomiting, hematochezia | Acute abdomen | N/A | N/A | Colon (unknown localization), jejunum | N/A | N/A | N/A | N/A | Yes | N/A | Unaffected | N/A | Yes | | N/A | Lymphocytes | No |  |
| Tempia-Caliera | 2003 | F | 64 |  | Abdominal pain, hematochezia | N/A | N/A | N/A | Ascending colon | Small veins | N/A | N/A | N/A | N/A | N/A | Unaffected | Thrombi of different age | N/A | | Hemorrhagic necrosis | Lymphocytes | N/A | ***(*) |
| Bando | 2003 | F | 43 |  | Abdominal pain, fever, other (pleurisy) | Acalculous cholecystitis | N/A | 1 month | Other (gallbladder) | N/A | Submucosal | N/A | N/A | Yes | N/A | Unaffected | Fresh thrombi | Patchy ischemic lesions of mucosa | | N/A | Lymphocytes, epitheloid cell | Yes | **** |
| Lavu | 2003 | F | 54 |  | Abdominal pain, hematochezia, diarrhea | N/A | 3 months | Approx. 17 months | Sigmoid, descending colon | Small and large | Mucosal | Yes | N/A | N/A | Yes | Unaffected | Yes | N/A | | N/A | Lymphocytes | N/A | ***** |
| Ordonez | 2004 | M | 62 |  | Abdominal pain, hematochezia | Acute abdomen, hemorrhagic shock | N/A | N/A | Cecum, ileum | N/A | N/A | Yes | Yes | N/A | N/A | Unaffected | Yes | Yes | | N/A | N/A | No | **** |
| Bao | 2005 | F | 25 |  | Abdominal pain, hematochezia, diarrhea, other (tenesmus) | N/A | 5 months | 5 months | Rectum, sigmoid colon | N/A | Submucosal, mesenterial | Yes | Yes | N/A | N/A | Unaffected | Thrombi of different age | N/A | | N/A | Mononuclear cells | N/A | ***** |
| Charron | 2005 | M | 71 |  | Abdominal pain, nausea, fever | N/A | Other (max. 2 days) | Approx. 1 week | Jejunum | N/A | Mesenterial | N/A | Yes | Yes | N/A | Unaffected | Yes | Ischemic mucosal injury | | N/A | Lymphocytes | N/A | ***** |
| Hu | 2005 | F | 72 |  | Abdominal pain, nausea, constipation, other (anorexia) | Acute abdomen | 5 days | N/A | Jejunum | N/A | Submucosal, mesenterial | No | Yes | N/A | N/A | Unaffected | Fresh thrombi | Yes | | Transmural hemorrhagic infarction | Eosinophils, lymphocytes, fibroblasts | N/A | ***** |
| Hu | 2005 | F | 75 |  | Abdominal pain, nausea, constipation, other (anorexia) | Acute abdomen | 6 days | N/A | Ileum | N/A | Submucosal, mesenterial | No | Yes | N/A | N/A | Unaffected | Fresh thrombi | Yes | | Transmural hemorrhagic infarction | Eosinophils, lymphocytes, fibroblasts | N/A |  |
| Hu | 2005 | F | 31 |  | Abdominal pain, nausea, constipation, other (anorexia) | N/A | 1 day | N/A | Cecum, appendix | N/A | Submucosal, mesenterial | No | Yes | N/A | N/A | Unaffected | Fresh thrombi | N/A | | Transmural hemorrhagic infarction | Eosinophils, lymphocytes, fibroblasts | N/A |  |
| Hu | 2005 | M | 68 |  | Abdominal pain, nausea, constipation, other (anorexia) | Acute abdomen | 3 weeks | N/A | Jejunum | N/A | Submucosal, mesenterial | No | Yes | N/A | N/A | Unaffected | Fresh thrombi | Yes | | Transmural hemorrhagic infarction | Eosinophils, lymphocytes, fibroblasts | N/A |  |
| Hu | 2005 | M | 53 |  | Abdominal pain, nausea, constipation, other (anorexia) | Acute abdomen | 3 days | N/A | Ileum | N/A | Submucosal, mesenterial | No | Yes | N/A | N/A | Unaffected | Fresh thrombi | Yes | | Transmural hemorrhagic infarction | Eosinophils, lymphocytes, fibroblasts, polymorphs | N/A |  |
| Knauer | 2005 | M | N/A |  | Abdominal pain, hematochezia | N/A | Other (few days) | N/A | Ascending colon, cecum | Medium | All layers, mesentery | N/A | Yes | N/A | N/A | Unaffected | Thrombi of different age | Ischemic mucosa | | N/A | Eosinophils, lymphocytes, plasma cells | N/A | **** |
| Canavan | 2007 | M | 32 |  | Abdominal pain, hematochezia, diarrhea | N/A | 5 days | N/A | Descending and transverse colon | N/A | All layers, particularly submucosal | N/A | Yes | N/A | N/A | Unaffected | N/A | N/A | | Mucosal necrosis | Lymphocytes | No | ***** |
| Mesina | 2007 | M | 63 |  | Other (acute abdomen) | Acute abdomen | N/A | N/A | Sigmoid colon | Small | Submucosal, mesenterial | No | Yes | N/A | No | Unaffected | Thrombi of different age | Transmural hemorrhagic infarction | | Epithelial necrosis | Eosinophils, lymphocytes, fibroblasts, polymorphs, plasma cells, mast cells | No | ***** |
| Perez-Corral | 2007 | M | 42 |  | Abdominal pain, diarrhea, weight loss | N/A | N/A | Min. 1 month | Ileum | Small, medium | N/A | Yes | No | N/A | Yes | Unaffected | No | N/A | | N/A | N/A | N/A | **** |
| Eryigit | 2008 | M | 39 |  | Abdominal pain, nausea, fever, constipation, other (anorexia) | N/A | 6 days | N/A | Ileum | Small, medium | Mesenterial | N/A | Yes | Yes | N/A | Unaffected | Yes | Yes | | Necrotic ileum | Lymphocytes, polymorphs | No | ***** |
| Tuncer | 2011 | M | 65 |  | Fever, hematochezia, other (proctalgia) | N/A | 2 weeks | N/A | Rectum, sigmoid colon | N/A | N/A | N/A | Yes | N/A | N/A | Unaffected | Yes | N/A | | Colon wall and mesentery | Polymorpho-Nuclear leucocytes | No | ***** |
| Miracle | 2013 | M | 34 |  | Abdominal pain, hematochezia | N/A | 3 months | N/A | Rectum, sigmoid colon, Descending colon, transverse colon | N/A | N/A | Yes | Yes | N/A | N/A | Unaffected | N/A | Lamina propria hemorrhage as early ischemic signs | | N/A | Neutrophils | N/A | ***** |
| Miracle | 2013 | M | 65 |  | Abdominal pain, diarrhea | N/A | Other (several months) | N/A | Rectum, sigmoid colon, descending colon | Small, medium | N/A | Yes | Yes | N/A | N/A | Unaffected | N/A | Mucosal ischemia | | N/A | Lymphocytes, plasma cells, neutrophils | N/A |  |
| Ayres | 2014 | M | 52 |  | Abdominal pain, diarrhea, weight loss, other (bloating) | N/A | 4 months | N/A | Sigmoid colon | N/A | N/A | Yes | N/A | N/A | Yes | Modest atherosclerosis | N/A | Yes | | N/A | N/A | N/A | ***** |
| Allali | 2018 | F | 64 |  | Abdominal pain, fever | N/A | N/A | N/A | Other (spleen) | Medium | N/A | N/A | N/A | N/A | Yes | Splenic artery thrombosis, intimal thickening due to early atherosclerosis | N/A | splenic ischemia | | N/A | Lymphocytes | N/A | **** |
| Yamada | 2018 | M | 32 |  | Abdominal pain, fever, hematochezia, diarrhea | N/A | N/A (3 years before: diagnosis UC due to hematochezia) | Approx. 1 month | Rectum, sigmoid colon, descending colon, transverse colon | Small | Submucosal, subserosal | Yes | N/A | N/A | Yes | Unaffected | Thrombi of different age | N/A | | N/A | N/A | N/A | **** |
| Matsuda | 2020 | M | 65 |  | Abdominal pain | N/A | N/A | Approx. 2 months | Rectum, sigmoid colon, descending colon | N/A | N/A | N/A | Yes | N/A | Yes | Unaffected | N/A | N/A | | N/A | N/A | N/A | **** |
| **IMHMV** | | | | | | | | | | | | | | | | | | |  | | | | |
| Genta | 1991 | M | 30 |  | Abdominal pain, hematochezia | N/A | 4 weeks | N/A | Sigmoid colon | 0.14–1 mm | Submucosal, muscularis propria, mesenterial | Yes | Yes | N/A | Yes | Modest intimal thickening in some arteries | Yes | Yes | | Ischemic necrosis of mucosa | No inflammation | N/A | ***** |
| Genta | 1991 | M | 38 |  | Abdominal pain, hematochezia, diarrhea, constipation | N/A | 2 months | N/A | Rectum, sigmoid colon, descending colon | 0.14–1mm | Submucosal, muscularis propria, mesenterial | Yes | Yes | N/A | Yes | Modest intimal thickening in some arteries | Yes | Yes | | Mucosal necrosis | No inflammation | N/A |  |
| Genta | 1991 | M | 25 |  | Abdominal pain, hematochezia, diarrhea, constipation | Acute abdomen | Approx. 6 months | Approx. 1 year | Rectum, sigmoid colon | 0.14–1mm | Submucosal, muscularis propria, mesenterial | Yes | Yes | N/A | Yes | Modest intimal thickening in some arteries | Yes | Yes | | Focal necrosis | No inflammation | N/A |  |
| Genta | 1991 | M | 67 |  | Abdominal pain, diarrhea, constipation | N/A | N/A | > 3 months | Sigmoid colon | 0.14–1mm | Submucosal, muscularis propria, mesenterial | Yes | Yes | N/A | Yes | Modest intimal thickening in some arteries | Yes | Yes | | Focal necrosis of mesocolic fat | No inflammation | N/A |  |
| Abu-Alfa | 1996 | M | 58 |  | Abdominal pain, hematochezia, diarrhea | N/A | N/A | > 1 year | Sigmoid colon | N/A | Submucosa, lamina propria, adventitia, mesocolon | Yes | N/A | N/A | Yes | Minority with subintimal thickening | Thrombi of different age | N/A | | Superficial necroses in areas of ulceration | No inflammation | N/A | ***** |
| Savoie | 1999 | M | 22 |  | Abdominal pain, hematochezia, diarrhea | N/A | 2 weeks | N/A | Rectum, sigmoid colon | N/A | N/A | Yes | N/A | N/A | N/A | Atherosclerosis | N/A | Yes | | N/A | N/A | N/A | ****(*) |
| De Hertogh | 2005 | M | 57 |  | Abdominal pain, diarrhea | N/A | N/A | > 10 months | Ascending colon, cecum, ileum | N/A | N/A | Yes | N/A | N/A | Yes | Unaffected | N/A | N/A | | Submucosal necrosis | No inflammation | N/A | ***** |
| Kao | 2005 | M | 38 |  | Abdominal pain, hematochezia, constipation, weight loss | Acute abdomen after 5 months | 2 months | 5 months | Rectum, sigmoid colon | N/A | N/A | Yes | N/A | N/A | Yes | Unaffected | Yes | Yes | | Ischemic necrosis of mucosa and bowel wall | N/A | N/A | ***** |
| Garcia-Castellanos | 2010 | F | 32 |  | Abdominal pain, hematochezia, diarrhea | Abdominal mass | 4 weeks | 3 months | Sigmoid colon | N/A | Mucosal, muscularis propria, serosal | Yes | N/A | N/A | N/A | Unaffected | N/A | Mucosal ischemia | | N/A | N/A | N/A | ***** |
| Chiang | 2012 | M | 60 |  | Abdominal pain, hematochezia, diarrhea, weight loss | N/A | 4 weeks | N/A | Rectum, sigmoid colon | N/A | N/A | Yes | N/A | N/A | Yes | Unaffected | N/A | N/A | | N/A | No inflammation | N/A | ***** |
| Korenblit | 2012 | M | 62 |  | Abdominal pain, hematochezia | N/A | 10 months | N/A | Total colon | Small, medium | Mucosal, submucosal, pericolic fat | Yes | N/A | N/A | Yes | Unaffected | N/A | Mucosal ischemia, chronic ischemia submucosal | | N/A | No inflammation | N/A | ***** |
| Lanitis | 2012 | M | 81 |  | Abdominal pain, constipation, weight loss, other (anorexia, abdominal distention) | Ascites | 6 months | N/A | Ileum | N/A | Lamina propria | Yes | N/A | N/A | N/A | Unaffected | N/A | N/A | | N/A | No inflammation | N/A | ***** |
| Feo | 2012 | F | 75 |  | Abdominal pain, hematochezia, diarrhea, weight loss, other (tenesmus) | N/A | 6 months | N/A | Rectum, sigmoid colon | N/A | N/A | Yes | N/A | N/A | N/A | Unaffected | N/A | Chronic ischemic injury | | N/A | No inflammation | N/A | ****(*) |
| Thomas | 2013 | M | 62 |  | Hematochezia, diarrhea | Abdominal mass | N/A | N/A | Rectum, sigmoid colon | N/A | N/A | Yes | N/A | N/A | N/A | N/A | N/A | N/A | | Fat necrosis | N/A | N/A | ****(*) |
| Laskaratos | 2014 | F | 62 |  | Abdominal pain, diarrhea | N/A | N/A | N/A | Ileum | N/A | N/A | Yes | N/A | N/A | N/A | N/A | N/A | N/A | | N/A | N/A | N/A | ***(*) |
| Sahara | 2015 | M | 76 |  | Abdominal pain, diarrhea | N/A | 1 year | 1 year | Sigmoid colon | N/A | Submucosal | Yes | N/A | N/A | Yes | Unaffected | Occasional hyalin thrombi | Ischemic mucosa | | N/A | Little lymphocytic infiltrate | N/A | ***** |
| Wangensteen | 2015 | F | 62 |  | Abdominal pain, hematochezia, diarrhea | N/A | Other (several months) | N/A | Rectum, sigmoid colon, descending colon | N/A | Mucosal, submucosal, subserosal | Yes | N/A | N/A | N/A | Unaffected | N/A | Ischemic ulceration | | N/A | N/A | N/A | ***** |
| Costa | 2016 | M | 47 |  | Abdominal pain, hematochezia, diarrhea, other (proctalgia, malaise) | N/A | 9 months | 9 months | Rectum, sigmoid colon | N/A | Submucosal, adventitia | Yes | Yes | N/A | Yes | Unaffected | Yes | Ischemic mucosa | | N/A | N/A | N/A | ***** |
| Guadagno | 2016 | F | 59 |  | Abdominal pain, diarrhea, weight loss | N/A | 6 months | N/A | Ileum | N/A | N/A | Yes | N/A | N/A | Yes | Unaffected | No | Ischemic mucosa | | N/A | No inflammation | N/A | ***** |
| Patel | 2016 | M | 65 |  | Abdominal pain, diarrhea, other (tenesmus) | N/A | 6 weeks | N/A | Rectum, sigmoid colon | N/A | N/A | Yes | N/A | N/A | N/A | Unaffected | N/A | Ischemic mucosa | | N/A | N/A | N/A | ****(*) |
| Yun | 2016 | M | 64 |  | Abdominal pain, hematochezia, diarrhea | N/A | 2 years | 2 years | Rectum, sigmoid colon, descending colon and transverse colon | Medium, large | N/A | Yes | N/A | N/A | Yes | Unaffected | Thrombosis of distal inferior mesenteric vein | N/A | | Fat necrosis | No inflammation | N/A | ***** |
| Song | 2017 | M | 59 |  | Abdominal pain, diarrhea, constipation, other (bloating) | N/A | 30 years | 30 years | Sigmoid, descending, transverse and ascending colon, ileum | N/A | N/A | Yes | N/A | N/A | Yes | Unaffected | N/A | No mucosal ischemia | | N/A | No inflammation | N/A | ***** |
| Yantiss | 2017 | M | 71 |  | Abdominal pain, hematochezia, diarrhea | N/A | Other (several months) | N/A | Rectum, sigmoid colon, descending colon | Medium, large | Submucosal, mesenteric fat | Yes | N/A | N/A | Yes | Unaffected | Yes | Yes | | N/A | No inflammation | N/A | **** |
| Yantiss | 2017 | M | 83 |  | Abdominal pain, hematochezia, diarrhea | N/A | Other (several months) | N/A | N/A | Medium, large | Submucosal, mesenteric fat | Yes | N/A | N/A | Yes | Unaffected | Yes | Yes | | N/A | No inflammation | N/A |  |
| Yantiss | 2017 | M | 83 |  | Hematochezia | Abdominal mass | Other (several months) | N/A | Rectum, sigmoid colon, descending colon | Medium, large | Submucosal, mesenteric fat | Yes | N/A | N/A | Yes | Unaffected | Yes | Yes | | N/A | No inflammation | N/A |  |
| Yantiss | 2017 | M | 63 |  | Hematochezia, weight loss | N/A | Other (several months) | N/A | Rectum, sigmoid colon, descending colon | Medium, large | Submucosal, mesenteric fat | Yes | N/A | N/A | Yes | Unaffected | Yes | Yes | | N/A | No inflammation | N/A |  |
| Yantiss | 2017 | M | 78 |  | Abdominal pain, hematochezia, diarrhea | N/A | Other (several months) | N/A | Rectum, sigmoid colon, descending colon | Medium, large | Submucosal, mesenteric fat | Yes | N/A | N/A | Yes | Unaffected | Yes | Yes | | N/A | No inflammation | N/A |  |
| Yantiss | 2017 | F | 73 |  | Hematochezia, weight loss | N/A | Other (several months) | N/A | Descending colon | Medium, large | Submucosal, mesenteric fat | Yes | N/A | N/A | Yes | Unaffected | Yes | Yes | | N/A | No inflammation | N/A |  |
| Yantiss | 2017 | M | 65 |  | Abdominal pain, hematochezia, diarrhea | N/A | Other (several months) | N/A | Rectum, sigmoid colon, descending colon | Medium, large | Submucosal, mesenteric fat | Yes | N/A | N/A | Yes | Unaffected | Yes | Yes | | N/A | No inflammation | N/A |  |
| Yantiss | 2017 | M | 64 |  | Abdominal pain, hematochezia, diarrhea | N/A | Other (several months) | N/A | Rectum, sigmoid colon, descending colon | Medium, large | Submucosal, mesenteric fat | Yes | N/A | N/A | Yes | Unaffected | Yes | Yes | | N/A | No inflammation | N/A |  |
| Yantiss | 2017 | M | 25 |  | Abdominal pain, diarrhea | N/A | Other (several months) | N/A | Sigmoid colon | Medium, large | Submucosal, mesenteric fat | Yes | N/A | N/A | Yes | Unaffected | Yes | Yes | | N/A | No inflammation | N/A |  |
| Yantiss | 2017 | M | 71 |  | Hematochezia | N/A | Other (several months) | N/A | Rectum, sigmoid colon, descending colon | Medium, large | Submucosal, mesenteric fat | Yes | N/A | N/A | Yes | Unaffected | Yes | Yes | | N/A | No inflammation | N/A |  |
| Abott | 2018 | M | 58 |  | Abdominal pain, hematochezia, diarrhea | N/A | > 4 weeks | N/A | Rectum, sigmoid, descending colon | N/A | N/A | Yes | N/A | N/A | N/A | Unaffected | N/A | Ischemic sigmoid colon | | N/A | N/A | N/A | ****(*) |
| Gonai | 2018 | M | 68 |  | Constipation, other (mucous stool, abdominal fullness) | N/A | N/A | N/A | Sigmoid, descending colon | N/A | Submucosal, subserosal | Yes | N/A | N/A | N/A | N/A | N/A | N/A | | N/A | No inflammation | N/A | ****(*) |
| Louie | 2018 | M | 45 |  | Hematochezia | N/A | N/A | N/A | Rectum, sigmoid colon | N/A | Mucosal, submucosal | Yes | N/A | N/A | Yes | Unaffected | N/A | N/A | | Transmural necrosis | N/A | N/A | **** |
| Louie | 2018 | F | 57 |  | Abdominal pain, nausea | N/A | N/A | N/A | Jejunum | Small, medium | Submucosal | Yes | N/A | N/A | Yes | N/A | N/A | N/A | | N/A | No inflammation | N/A |  |
| Anderson | 2019 | 6 M, 2 F | Median 62.5 (22–75) |  | Abdominal pain (7/8), hematochezia (4/8), diarrhea (5/8) | N/A | Median 2 months (1–6 months) | N/A | Sigmoid colon (6/8) | N/A | Mucosal, submucosal, extramural | Yes | N/A | N/A | N/A | N/A | N/A | Yes | | N/A | N/A | N/A | ***(*) |
| Chudy-onwugaje | 2020 | M | 54 |  | Abdominal pain, diarrhea, weight loss | N/A | 4 weeks | 4 months | N/A | N/A | N/A | Yes | N/A | N/A | N/A | N/A | N/A | N/A | | N/A | N/A | N/A | ****(*) |
| Kelly | 2020 | M | 53 |  | Abdominal pain, hematochezia, weight loss, other (tenesmus) | N/A | 3 months | N/A | Rectum, sigmoid colon, descending colon | N/A | Lamina propria | Yes | N/A | N/A | Yes | Unaffected | Yes | N/A | | Mucosal necrosis | N/A | N/A | ***** |
| Martin | 2020 | M | 63 |  | Diarrhea, weight loss, other (incontinence) | N/A | 3 months | 5 months | Sigmoid colon | Capillaries, small | Submucosal | Yes | N/A | Yes | Yes | N/A | Yes | Yes | | N/A | N/A | N/A | ***** |
| Al Ansari | 2021 | M | 63 |  | Abdominal pain, diarrhea | N/A | N/A | > 1 month | Sigmoid, descending colon | N/A | Mucosal, lamina propria, pericolic fat | Yes | N/A | N/A | Yes | N/A | Yes | Yes | | N/A | N/A | N/A | **** |
| Yamada | 2021 | F | 81 |  | Abdominal pain, nausea, vomiting | N/A | N/A | N/A | Ileum | N/A | Mucosal, lamina propria, subserosal | Yes | N/A | No | Yes | Unaffected | N/A | N/A | | N/A | Lymphocytes, plasma cells | N/A | **** |

EP, enterocolic phlebitis; MIVOD, mesenteric inflammatory veno-occlusive disease; IMHMV, idiopathic myointimal hyperplasia of mesenteric veins; M, male; F, female; N/A, not available; GI-tract, gastrointestinal tract; UC, ulcerative colitis.
